# Impact of sarcopenia on postoperative clinical outcomes in patients with cervical spondylotic myelopathy undergoing anterior cervical discectomy and fusion

**DOI:** 10.1186/s40001-025-03444-z

**Published:** 2025-11-13

**Authors:** Yan Gong, Xiangyu Long, Hao Liu, Jiahui He, Yanchi Gan, Yixuan Jiang, Hang Zhuo, Zelin Zhou, Kai Tang, De Liang, Xiaobing Jiang, Zhaojun Cheng, Jintao Liu

**Affiliations:** 1 Suzhou TCM Hospital Affiliated With Nanjing University of Chinese Medicine, Suzhou, 215009 China; 2 Nanjing University of Chinese Medicine, Nanjing, 210023 China; 3 The Second Affiliated Hospital of Guangzhou Medical University, Guangzhou, 510260 China; 4 The First Affiliated Hospital of Guangzhou University of Chinese Medicine, Guangzhou, 510405 China; 5 The Affiliated TCM Hospital of Guangzhou Medical University, Guangzhou, 510120 China; 6 Ruikang Hospital Affiliated With Guangxi University of Chinese Medicine, Guangxi, 530000 China; 7 Faculty of Chinese Medicine, Macau University of Science and Technology, State Key Laboratory of Quality Research in Chinese Medicine, Taipa, 999078 Macao China

**Keywords:** Sarcopenia, Anterior cervical discectomy and fusion, Cervical spondylotic myelopathy, Sagittal parameters, Clinical efficacy

## Abstract

**Objectives:**

This study investigated the impact of sarcopenia diagnosed on the basis of the neck muscle index on the clinical efficacy of anterior cervical discectomy and fusion (ACDF) surgery in patients with cervical spondylotic myelopathy (CSM) 2 years after surgery.

**Methods:**

A retrospective analysis of the clinical data of 93 patients who underwent ACDF surgery for CSM between March 2019 and May 2023 was conducted. Patients meeting the sarcopenia diagnostic criteria of the C3 paraspinal muscle index (C3MI) for males and the sternocleidomastoid muscle index (SCMI) for females were divided into a sarcopenia group (38 patients) and a nonsarcopenia group (55 patients). Differences in the perioperative indicators, imaging data, and clinical efficacy scores between the two groups were compared.

**Results:**

Both groups of patients successfully underwent surgery, and no severe complications occurred postoperatively. In terms of baseline data and perioperative indicators, there were no statistically significant differences between the two groups in terms of sex, age, body mass index (BMI), intraoperative blood loss, surgical duration, preoperative odontoid incidence angle (OI), preoperative cervical spine range of motion (ROM), or preoperative adjacent segment ROM of the surgical segment (*P* > 0.05). Compared with preoperative values, both groups of patients demonstrated significant improvements in the T1 slope (T1s), C2‒C7 Cobb angle, C0‒C2 Cobb angle, sagittal segmental angle (SSA), and average surgical segment disc height (ASDH) immediately after surgery (*P* < 0.05). In terms of clinical functional scores, both groups of patients showed significant improvements in the Japanese Orthopedic Association (JOA) score, visual analog scale (VAS) score, and neck disability index (NDI) score immediately after surgery and at the 2-year follow-up compared with the preoperative scores (*P* < 0.05). The VAS score and NDI score at 2-year post-surgery, as well as the VAS score immediately postsurgery, were significantly worse in the sarcopenia group than in the nonsarcopenia group (*P* < 0.05). In addition, there were no statistically significant differences in cervical spine parameters or changes in these parameters between the two groups postoperatively (*P* > 0.05).

**Conclusions:**

Sarcopenia can have an adverse effect on pain relief and functional recovery in CSM patients undergoing ACDF surgery over a 2-year postoperative period. Clinicians should prioritize the assessment and intervention of sarcopenia in such patients to improve their long-term clinical outcomes following surgery.

## Introduction

Cervical spondylotic myelopathy (CSM) is one of the most severe types of cervical degenerative disease. It often causes symptoms such as limb sensory and motor dysfunction and abnormal gait due to compression of the spinal cord by cervical disc herniation and osteophyte formation, seriously affecting the quality of life of patients [1, 2]. Anterior cervical discectomy and fusion (ACDF) is a classic surgical procedure for treating CSM. Directly decompressing and fusing the segment can effectively improve spinal cord function and has been widely used in clinical practice [3]. However, postoperative efficacy is influenced by a variety of factors. In addition to surgical technique and disease severity, the patient's overall physical condition is receiving increasing attention [4].

Sarcopenia, an age-related systemic skeletal muscle disease, is characterized by a decrease in muscle mass, strength, and function. Its incidence rate is increasing annually in the elderly population [5]. Recent studies have shown that sarcopenia involves not only physiological degeneration associated with aging but also the prognosis of various chronic diseases. In particular, in the field of spinal surgery, its impact on postoperative recovery from degenerative spinal diseases has gradually become a focus of research [6, 7]. Previous studies have shown that sarcopenia may affect pain relief and functional recovery after surgery for lumbar degenerative diseases [8, 9], but research on how sarcopenia affects the medium-to-long-term (e.g., 2 years after surgery) clinical efficacy in CSM patients who have undergone ACDF surgery is lacking. The specific mechanism of action and clinical significance of sarcopenia remain unclear.

Therefore, in this study, a retrospective analysis of clinical data from CSM patients who underwent ACDF treatment was conducted, and the preoperative cervical skeletal muscle index was used as the criterion to divide patients into sarcopenia and nonsarcopenia groups. This study compared perioperative indicators and clinical outcomes at 2 years post-operatively between the two groups, aiming to explore the impact of sarcopenia on the medium-to-long-term outcomes of ACDF surgery in CSM patients. This research provides a reference for optimizing preoperative assessments and developing targeted intervention strategies to improve patient outcomes.

## Materials and methods

### Participants

This study included 93 patients with short-segment CSM who underwent ACDF surgery at the First Affiliated Hospital of Guangzhou University of Chinese Medicine from March 2019 to May 2023, with an average postoperative follow-up duration of 2 years. As a single-center retrospective study, this sample size accurately represents the entire cohort of patients with complete follow-up data available during this period, which is consistent with the scale of similar studies in the field of spine surgery. The inclusion criteria were as follows: ① clinically diagnosed with CSM involving two or fewer segments on the basis of clinical symptoms, physical examination, and imaging studies (such as cervical spine *X*-rays, CT, or MRI) and ② inadequate response to standardized conservative treatment, with symptoms severely impacting daily activities. The exclusion criteria for patients were as follows: ① presence of congenital cervical spine deformities (e.g., congenital vertebral fusion); ② history of cervical trauma (e.g., vertebral fractures, joint dislocations, etc.) or prior history of cervical organic injuries; ③ long-term regular use of aspirin, clopidogrel, etc., and inability to discontinue medication according to standard protocols prior to surgery; ④ concurrent severe underlying diseases of vital organs such as the heart, liver, or kidneys that were deemed unable to tolerate surgery following multidisciplinary assessment by the anesthesiology and surgical departments; and ⑤ history of cervical spine surgery (e.g., cervical fusion surgery, discectomy for herniated disc, etc.). This study protocol was approved by the Institutional Review Board of the First Affiliated Hospital of Guangzhou University of Chinese Medicine. All patients signed informed consent forms prior to the study and explicitly agreed to the use of their imaging data for academic research and related publications.

### Surgical procedure

All surgeries in this study were performed by the same lead surgeon and their team following standardized procedures. Patients underwent routine tracheal displacement training 1–2 days prior to surgery to adapt to the surgical requirements. After general anesthesia and tracheal intubation, the patient was placed in the supine position with the head fixed and the shoulders moderately tractioned downward. A transverse surgical incision was made on the right side of the neck, and the skin and subcutaneous tissue were dissected layer by layer along the incision. The dissection is then continued via blunt dissection techniques to reach the anterior intervertebral space. The trachea and esophagus are pulled to the left and properly retracted, and the longus colli muscle and anterior vertebral fascia are further dissected to fully expose the anterior longitudinal ligament. The target intervertebral space is marked with a positioning needle, and its position is confirmed to be accurate via *C*-arm *X*-ray fluoroscopy technology. A Caspar retractor was used to open the target intervertebral space, the intervertebral disc tissue was completely removed, the dural sac was examined, and adequate decompression was performed until the dural sac returned to its normal bulging state. After the cartilage tissue at the endplates of the upper and lower vertebrae was scraped, an intervertebral fusion device filled with bone chips was implanted into the intervertebral space. All remaining target intervertebral spaces are treated via the same procedure, followed by fixation of the implanted internal device with screws. *C*-arm fluoroscopy is performed again to confirm that the positions of the internal device and screws meet satisfactory standards, after which the surgical field is thoroughly irrigated. Hemostasis is achieved via an electrosurgical knife, followed by placement of a drainage tube. The surgical incision was then closed in layers, disinfected, and covered with sterile gauze. Postoperative vital sign monitoring was performed on the patient. Antibiotics were administered for 24 h according to the standard protocol. The drainage tube was removed 1–2 days post-operatively. The patient must wear a cervical collar for 8–12 weeks. On the basis of the patient's postoperative recovery status, they are guided to gradually initiate cervical functional exercises.

### Radiographic analyses and data collection

**Fig. 1:**
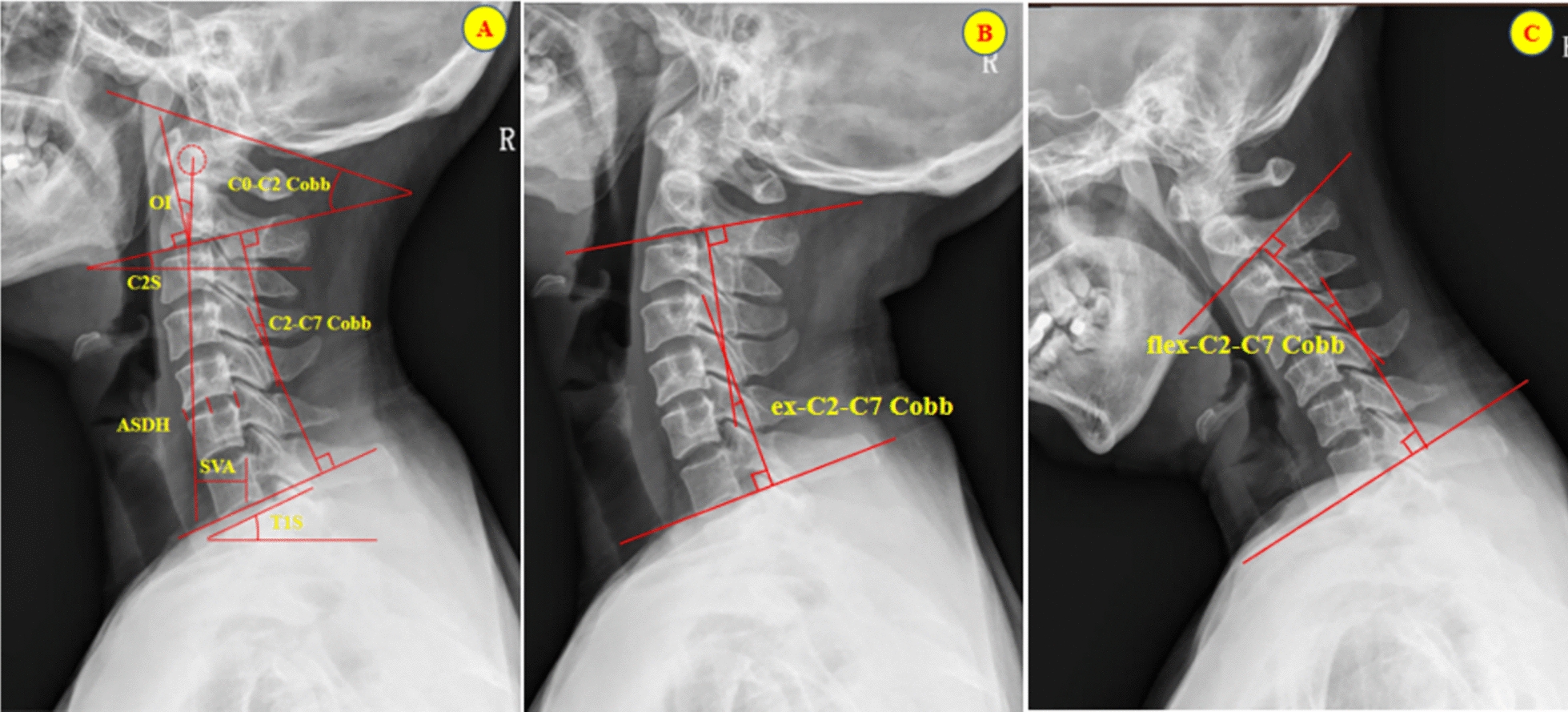
Measurement of cervical spine parameters in sagittal plane images of cervical spine *X*-rays. **A** Measurement of the C2‒C7 Cobb angle, C0‒C2 Cobb angle, SVA, T1s, C2s, and OI; **B** C2‒C7 Cobb angle in cervical spine *X*-rays taken during hyperextension; **C** C2‒C7 Cobb angle in cervical spine *X*-rays taken during hyperflexion

Baseline data and perioperative data, including age, sex, surgical duration, intraoperative blood loss, and body mass index (BMI), were recorded for the two groups of patients. Preoperative and postoperative lateral and dynamic *X*-ray images of the cervical spine were collected to assess sagittal plane-related parameters of the cervical spine. Among these, odontoid incidence (OI) refers to the angle formed between the midline vertical line of the lower endplate of C2 and the line extending from the midpoint of the lower endplate of C2 to the center of the odontoid process [10]. The C2‒C7 Cobb angle is the angle between the vertical line from the inferior endplate of the C2 vertebral body and the vertical line from the inferior endplate of the C7 vertebral body. The C0‒C2 Cobb angle is the angle between McRae’s line and the inferior endplate of the C2 vertebral body. The C2‒C7 sagittal vertical axis (SVA) is the distance from the center of the C2 vertebral body to the perpendicular line to the posterior superior angle of the C7 vertebral body. The T1 slope angle is the angle between the horizontal line and the line parallel to the superior endplate of the T1 vertebral body. The C2 slope angle is the angle between the horizontal line and the line parallel to the inferior endplate of the C2 vertebral body. The surgical segment anterior angle (SSA) refers to the angle between the upper endplate of the superior vertebra and the lower endplate of the inferior vertebra within the surgical segment; the surgical segment intervertebral disc height (ASDH) is the average of the anterior, middle, and posterior heights of the intervertebral disc; simultaneously, the range of motion (ROM) data of the superior and inferior vertebrae within the surgical segment (upper and lower SSA–ROM) are extracted from dynamic cervical radiographs. Postoperative recovery outcomes were evaluated via the Japanese Orthopedic Association (JOA) score, cervical visual analog scale (VAS), and neck disability index (NDI). JOA scores, NDI scores, and VAS scores were measured at baseline, immediate postoperative review, and at the 2-year post-operative follow-up. All the results were jointly assessed by two senior clinicians. The specific measurement methods for these indicators are shown in Fig. 1.

**Fig. 2:**
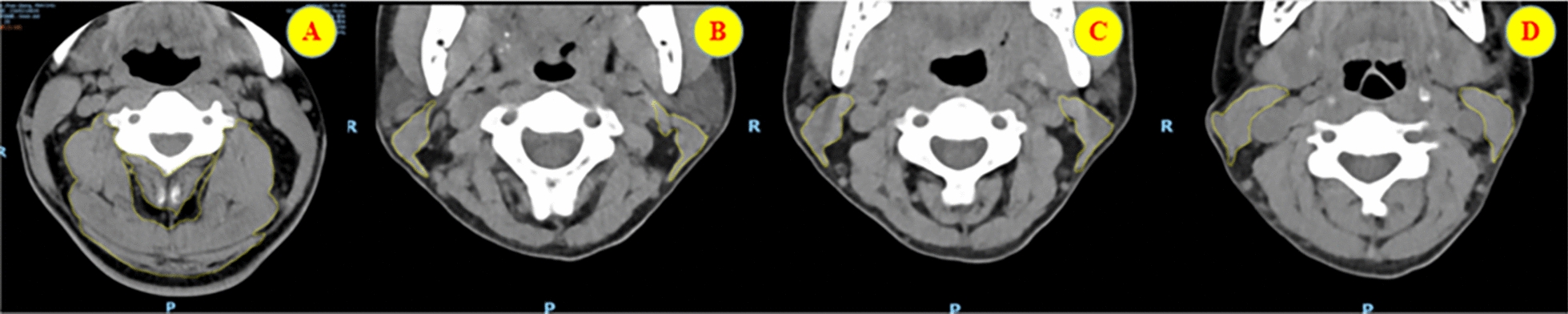
**A** Muscles measured at the C3 level in male patients include the neck rotator muscles, trapezius muscles, longus capitis muscles, semispinalis capitis muscles, splenius capitis muscles, and trapezius muscles. **B**, **C**, and **D** Areas of the sternocleidomastoid muscles in the C2, C3, and C4 cross-sections of female patients, respectively

This study diagnosed sarcopenia based on the specific neck muscle mass index established by Ufuk et al. [11], defined as a cervical paraspinal muscle index (C3MI) ≤ 9.3 cm^2^/m^2^ in males or a sternocleidomastoid muscle index (SCMI) ≤ 1.5 cm^2^/m^2^ in females, combined with a physical status (PS) score > 1. It should be noted that this standard quantifies muscle mass only and does not incorporate assessments of muscle strength (e.g., grip strength) or physical function (e.g., walking speed), differing from the comprehensive diagnostic framework recommended by the European Working Group on Sarcopenia in the Elderly (EWGSOP2). The rationale for adopting this criterion is as follows: first, as a retrospective study, systematic access to routine preoperative functional assessment data for patients was not feasible. Second, an increasing number of spinal surgery studies indicate that localized muscle mass correlates more strongly and directly with spinal disease prognosis than systemic indicators [12–14]. The cervical muscle group is central to maintaining dynamic stability of the cervical spine, and its atrophy is directly related to the pathological progression of cervical spondylosis and recovery following ACDF. This study employed the aforementioned diagnostic criteria to assess patients' sarcopenia status using ImageJ software. Based on the measurement results of the aforementioned indicators, patients were categorized into a sarcopenia group (38 cases, including 17 males and 21 females) and a non-sarcopenia group (55 cases, including 22 males and 33 females). A schematic diagram illustrating the measurement of relevant muscle cross-sectional areas is shown in Fig. 2.

### Reliability analysis of imaging area

To ensure the reliability and repeatability of cervical muscle cross-sectional area measurements, we conducted intra-rater and inter-rater reliability assessments. Two spinal surgeons (Surgeon A and Surgeon B), unaware of patient group assignments, independently measured preoperative images (measuring the total area of paracervical muscles at the C3 level for males, and the area of the sternocleidomastoid muscle at the C2–C4 levels for females, then averaging the results). To assess intra-rater reliability, Researcher A randomly selected 30 patients (12 males, 18 females) from the total sample for repeat measurements 4 weeks after the initial assessment. To evaluate inter-rater reliability, Researcher B independently measured the same 30 patients' images. Intraclass correlation coefficients (ICC) using a two-factor random effects model were employed to assess absolute agreement.

### Statistical analysis

This study utilized SPSS 27.0 statistical software for data analysis and GraphPad Prism 9.5 software for creating statistical graphs. Continuous variables are expressed as the means ± standard deviations; qualitative data were analyzed via the chi-square test; differences between preoperative and postoperative indicators were compared via the paired *t* test; and differences in cervical sagittal plane parameters and various scores between the two groups of patients were compared via the independent samples *t* test. The reliability of neck muscle measurements was assessed using the ICC. *P* < 0.05 indicated a statistically significant difference.

## Results

### Reliability analysis

The reliability analysis revealed that both intra-rater and inter-rater ICC values for all neck muscle measurements exceeded 0.960 (*P* < 0.001, Table 1), with 95% confidence intervals demonstrating excellent precision. This indicates exceptional reproducibility of the measurement method in this study and highly reliable data quality for core indicators.

**Table 1:** Intra- and inter-rater reliability of cervical muscle cross-sectional area measurements

Measurement Indicator	Intra-rater			Inter-rater		
	ICC value	95% CI	*P* value	ICC value	95% CI	*P* value
Male C3MI (*n* = 12)	0.960	[0.868, 0.988]	< 0.001	0.974	[0.908, 0.992]	< 0.001
Female SCMI-C2 (*n* = 18)	0.992	[0.978, 0.997]	< 0.001	0.989	[0.971, 0.996]	< 0.001
Female SCMI-C3 (*n* = 18)	0.993	[0.981, 0.997]	< 0.001	0.985	[0.959, 0.994]	< 0.001
Female SCMI-C4 (*n* = 18)	0.979	[0.945, 0.992]	< 0.001	0.994	[0.985, 0.998]	< 0.001

C3MI, C3 paraspinal muscle index; SCMI, sternocleidomastoid muscle index; ICC, intraclass correlation coefficient; CI, confidence interval

### Patient demographics

This study included a total of 93 patients with CSM, including 38 patients in the sarcopenia group and 55 patients in the nonsarcopenia group. The mean age of patients in the sarcopenia group was 63.79 ± 5.53 years, whereas the mean age of patients in the nonsarcopenia group was 61.60 ± 8.28 years. There were no statistically significant differences between the two groups in terms of baseline characteristics, including age, sex, BMI, surgical duration, intraoperative blood loss, preoperative OI, preoperative ROM, or preoperative upper SSA–ROM and lower SSA–ROM (*P* > 0.05, Table 2).

**Table 2:** Baseline characteristics of the population

Parameters	Sarcopenia (*n* = 38)	Nonsarcopenia (*n* = 55)	*P* value
Age (years)	63.79 ± 5.53	61.60 ± 8.28	0.158
Gender (*n*, %)			0.649
-Female	21 (55.26)	33 (60.00)	
-Male	17 (44.74)	22 (40.00)	
BMI	23.21 ± 2.84	23.37 ± 3.01	0.806
Operation Duration (min)	130.79 ± 34.16	126.55 ± 35.07	0.563
Blood Loss (mL)	36.31 ± 34.67	30.18 ± 19.20	0.277
OI (°)	13.38 ± 3.85	14.12 ± 4.60	0.419
ROM (°)	33.69 ± 18.40	30.18 ± 15.89	0.329
Upper SSA–ROM (°)	9.00 ± 5.07	8.12 ± 5.50	0.437
Lower SSA–ROM (°)	9.21 ± 4.50	9.75 ± 5.60	0.624

### Preoperative and postoperative comparisons of the cervical parameters of patients in the two groups

Compared with preoperative values, patients in the sarcopenia group showed significant improvements in the T1s, C2‒C7 Cobb angle, C0‒C2 Cobb angle, C2s, SSA, and ASDH immediately postoperatively (*P* < 0.05); patients in the nonsarcopenia group also showed significant improvements in the T1s, C2‒C7 Cobb angle, C0‒C2 Cobb angle, SSA, and ASDH immediately postoperatively (*P* < 0.05, Table 3).

**Table 3:** Comparison of intragroup preoperative‒postoperative differences in cervical spine parameters between the two groups of patients

Group	Parameters	Preoperation	Postoperation	*P* value
Sarcopenia	SVA (mm)	19.88 ± 12.76	20.87 ± 11.37	0.547
	T1s (°)	20.86 ± 7.24	27.38 ± 6.96	< 0.001
	C0–2 Cobb (°)	31.12 ± 6.59	22.99 ± 6.25	< 0.001
	C2–7 Cobb (°)	9.47 ± 6.17	17.48 ± 7.12	< 0.001
	C2s (°)	7.30 ± 4.31	10.36 ± 6.01	0.004
	SSA (°)	6.09 ± 4.56	10.67 ± 5.02	< 0.001
	ASDH (mm)	5.80 ± 1.20	8.93 ± 1.19	< 0.001
Nonsarcopenia	SVA (mm)	22.17 ± 10.64	20.53 ± 11.20	0.305
	T1s (°)	22.62 ± 7.20	27.16 ± 7.43	< 0.001
	C0–2 Cobb (°)	32.45 ± 6.49	23.41 ± 6.13	< 0.001
	C2–7 Cobb (°)	9.23 ± 5.31	17.23 ± 7.28	< 0.001
	C2s (°)	8.59 ± 5.12	10.41 ± 6.15	0.06
	SSA (°)	5.65 ± 4.15	10.92 ± 4.86	< 0.001
	ASDH (mm)	5.75 ± 1.16	9.01 ± 1.34	< 0.001

### Comparison of preoperative, immediate postoperative, and 2-year post-operative clinical scores between the two groups of patients

**Fig. 3:**
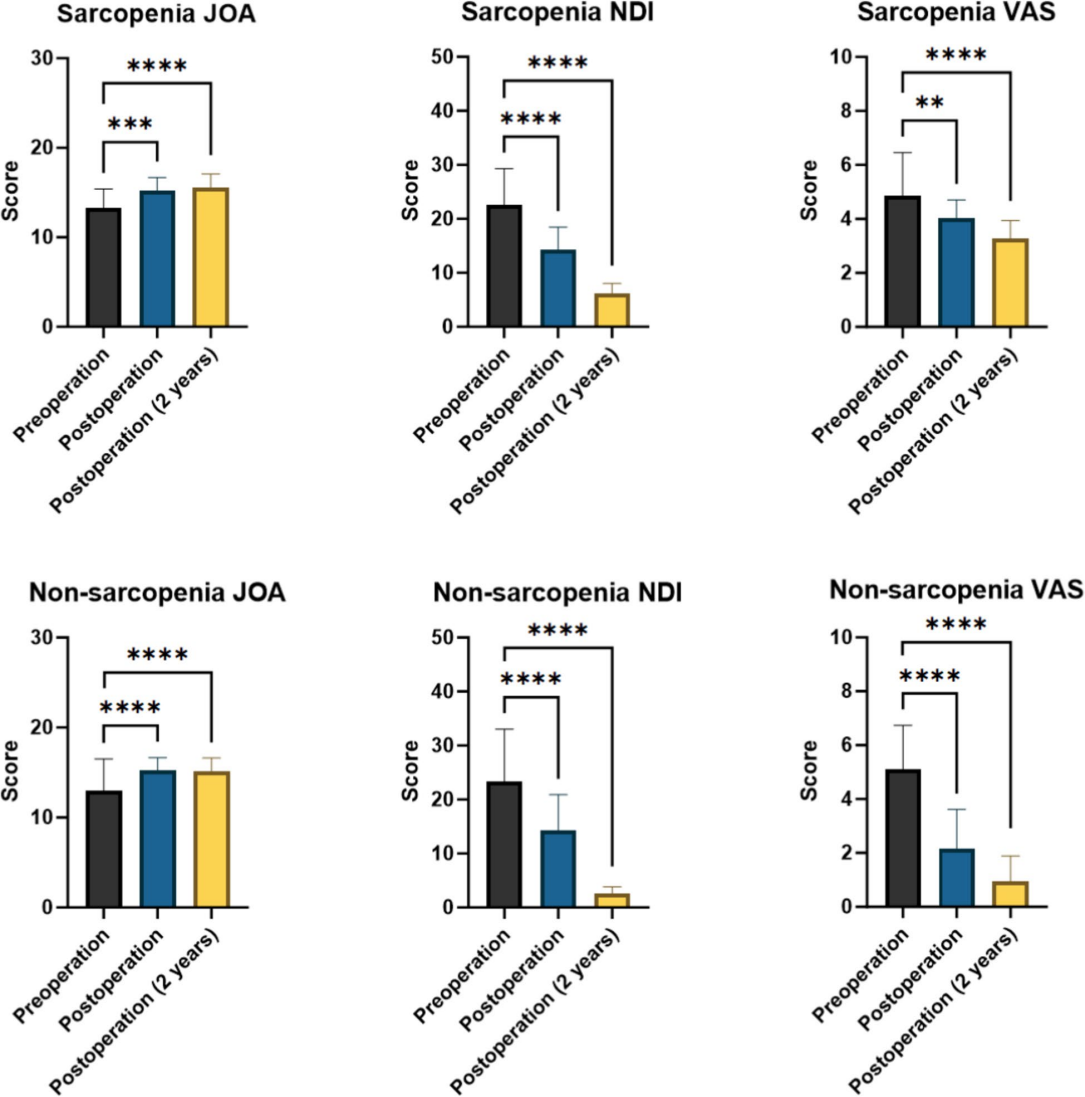
Comparison of JOA, NDI, and VAS scores between the two groups at preoperative, immediate postoperative, and 2-year postoperative follow-up time points

Compared with preoperative patients, both groups of patients demonstrated significant improvements in JOA, NDI, and VAS scores immediately after surgery and at the 2-year follow-up (*P* < 0.05; Table 4). Figure 3 not only visually demonstrates the improvement in the clinical outcomes of each group of patients but also further confirms the classic efficacy of ACDF surgery in alleviating clinical symptoms in patients with CSM.

**Table 4:** Comparison of preoperative, immediately postoperative, and 2-year post-operative clinical scores between the two patient groups

Group	Score	Preoperation	Postoperation	Post 2 years	*P* value (pre and post)	*P* value (pre and post 2)
Sarcopenia	JOA	13.24 ± 2.17	15.24 ± 1.46	15.53 ± 1.56	< 0.001	< 0.001
	NDI	22.55 ± 6.75	14.29 ± 4.17	6.13 ± 1.91	< 0.001	< 0.001
	VAS	4.87 ± 1.60	4.03 ± 0.68	3.29 ± 0.65	0.004	< 0.001
Nonsarcopenia	JOA	12.96 ± 3.55	15.24 ± 1.43	15.09 ± 1.53	< 0.001	< 0.001
	NDI	23.29 ± 9.74	14.36 ± 6.52	2.55 ± 1.29	< 0.001	< 0.001
	VAS	5.09 ± 1.65	2.16 ± 1.45	0.93 ± 0.96	< 0.001	< 0.001

### Comparison of sagittal plane parameters of the cervical spine between the two groups

There were no statistically significant differences between the sarcopenia group and the nonsarcopenia group in terms of T1s, C2‒C7 Cobb angle, C0‒C2 Cobb angle, cSVA, C2s, SSA, or ASDH preoperatively and immediately postoperatively (*P* > 0.05, Table 5).

**Table 5:** Intergroup comparisons of the sagittal plane parameters of the cervical spine in the two groups of patients

Parameters	Sarcopenia (*n* = 38)	Nonsarcopenia (*n* = 55)	*P* value
C2–C7cobb (°)			
Preoperation	9.47 ± 6.17	9.23 ± 5.31	0.841
Postoperation	17.48 ± 7.12	17.23 ± 7.28	0.865
C0–C2cobb (°)			
Preoperation	31.12 ± 6.59	32.45 ± 6.49	0.337
Postoperation	22.99 ± 6.25	23.41 ± 6.13	0.747
cSVA (mm)			
Preoperation	19.88 ± 12.76	22.17 ± 10.64	0.349
Postoperation	20.87 ± 11.37	20.53 ± 11.20	0.885
Tl slope (°)			
Preoperation	20.86 ± 7.24	22.62 ± 7.20	0.250
Postoperation	27.38 ± 6.96	27.16 ± 7.43	0.886
C2 slope (°)			
Preoperation	7.30 ± 4.31	8.59 ± 5.12	0.209
Postoperation	10.36 ± 6.01	10.41 ± 6.15	0.967
SSA (°)			
Preoperation	6.09 ± 4.56	5.65 ± 4.15	0.628
Postoperation	10.67 ± 5.02	10.92 ± 4.86	0.808
ASDH (mm)			
Preoperation	5.80 ± 1.20	5.75 ± 1.16	0.840
Postoperation	8.93 ± 1.19	9.01 ± 1.34	0.752

### Intergroup comparisons of JOA, NDI, and VAS scores preoperatively, immediately postoperatively, and at 2-year follow-up

**Fig. 4:**
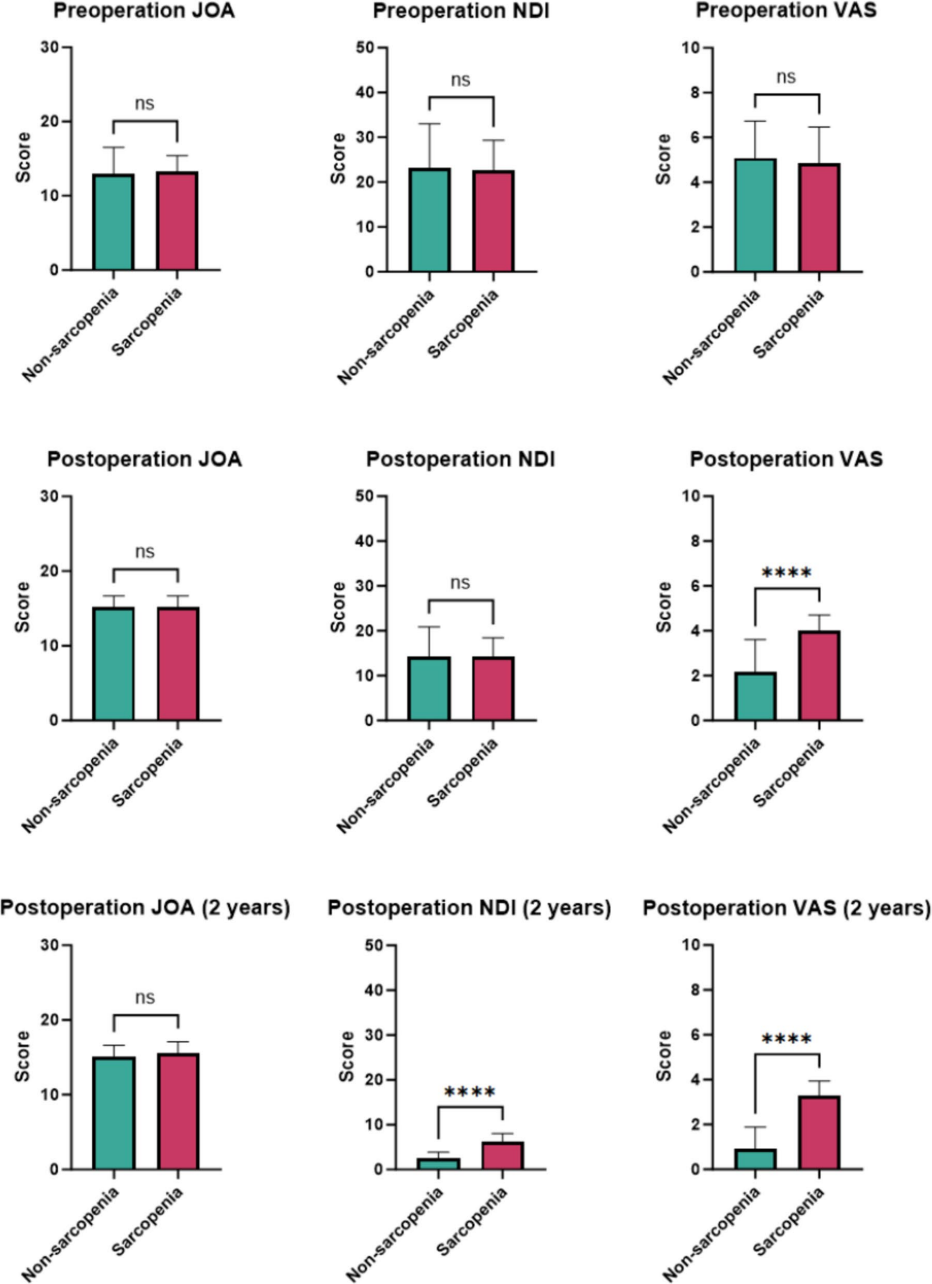
Comparison of JOA, NDI, and VAS scores between the two patient groups (preoperative, immediate postoperative, and 2-year postoperative follow-up)

There were no statistically significant differences between the two groups in terms of JOA scores at immediate postoperative and 2-year follow-up, preoperative and immediate postoperative NDI scores, or preoperative VAS scores (*P* > 0.05). However, there were statistically significant differences between the two groups in terms of NDI scores at the 2-year post-operative follow-up and VAS scores at both the immediate postoperative period and the 2-year postoperative follow-up (*P* < 0.05, Table 6). Figure 4 clearly shows the intergroup comparisons of the clinical scores at different timepoints between the two groups. In summary, sarcopenia has an impact on postoperative pain relief and long-term functional recovery in patients with CSM. As shown in Table 6, at the 2-year postoperative follow-up, the VAS score in the sarcopenia group was significantly higher than that in the nonsarcopenia group (3.29 ± 0.65 vs. 0.93 ± 0.96, *P* < 0.001), with an absolute difference of 2.36 points between the two groups. According to a recent systematic review of ACDF literature, the MCID threshold for neck VAS scores ranges from 2.5 to 3.1 points [15]. The observable between-group difference (2.36 points) is already very close to the lower limit of this range, indicating clear clinical significance.

**Table 6:** Intergroup comparisons of JOA, NDI, and VAS scores (preoperative, immediate postoperative, and 2-year postoperative follow-up)

Parameters	Sarcopenia (*n* = 38)	Nonsarcopenia (*n* = 55)	*P* value
JOA (score)			
Preoperation	13.24 ± 2.17	12.96 ± 3.55	0.674
Postoperation	15.24 ± 1.46	15.24 ± 1.43	0.999
Post 2 years	15.53 ± 1.56	15.09 ± 1.53	0.184
NDI (score)			
Preoperation	22.55 ± 6.75	23.29 ± 9.74	0.687
Postoperation	14.29 ± 4.17	14.36 ± 6.52	0.951
Post 2 years	6.13 ± 1.91	2.55 ± 1.29	< 0.001
VAS (score)			
Preoperation	4.87 ± 1.60	5.09 ± 1.65	0.518
Postoperation	4.03 ± 0.68	2.16 ± 1.45	< 0.001
Post 2 years	3.29 ± 0.65	0.93 ± 0.96	< 0.001

### Improvements in pre- and immediately postoperative parameters in the two groups of patients

There was no statistically significant difference in improvement in the immediate postoperative cSVA, T1s, C0‒C2 Cobb angle, C2‒C7 Cobb angle, C2s, SSA, or ASDH compared with the preoperative values between the two groups of patients (*P* > 0.05, Table 7). These findings suggest that sarcopenia has no significant effect on the improvement of cervical sagittal parameters after ACDF in patients with CSM.

**Table 7:** Improvements in sagittal parameters preoperative and immediate postoperative in the two patient groups

Parameters		Sarcopenia (*n* = 38)	Nonsarcopenia (*n* = 55)	*P* value
Post (Preop minus Postop)	∆cSVA	− 0.99 ± 10.05	1.65 ± 11.79	0.264
	∆T1s	− 6.52 ± 6.21	− 4.54 ± 6.19	0.133
	∆C0–2 Cobb	8.13 ± 4.26	9.04 ± 6.48	0.451
	∆C2–7 Cobb	− 8.02 ± 6.22	− 8.00 ± 6.69	0.990
	∆C2s	− 3.05 ± 6.17	− 1.82 ± 7.04	0.387
	∆SSA	− 4.58 ± 4.40	− 5.27 ± 4.53	0.463
	∆ASDH	− 3.13 ± 1.44	− 3.27 ± 1.23	0.626

### Preoperative vs. immediate postoperative and 2-year postoperative follow-up: improvements in JOA, NDI, and VAS scores in both patient groups

**Fig. 5:**
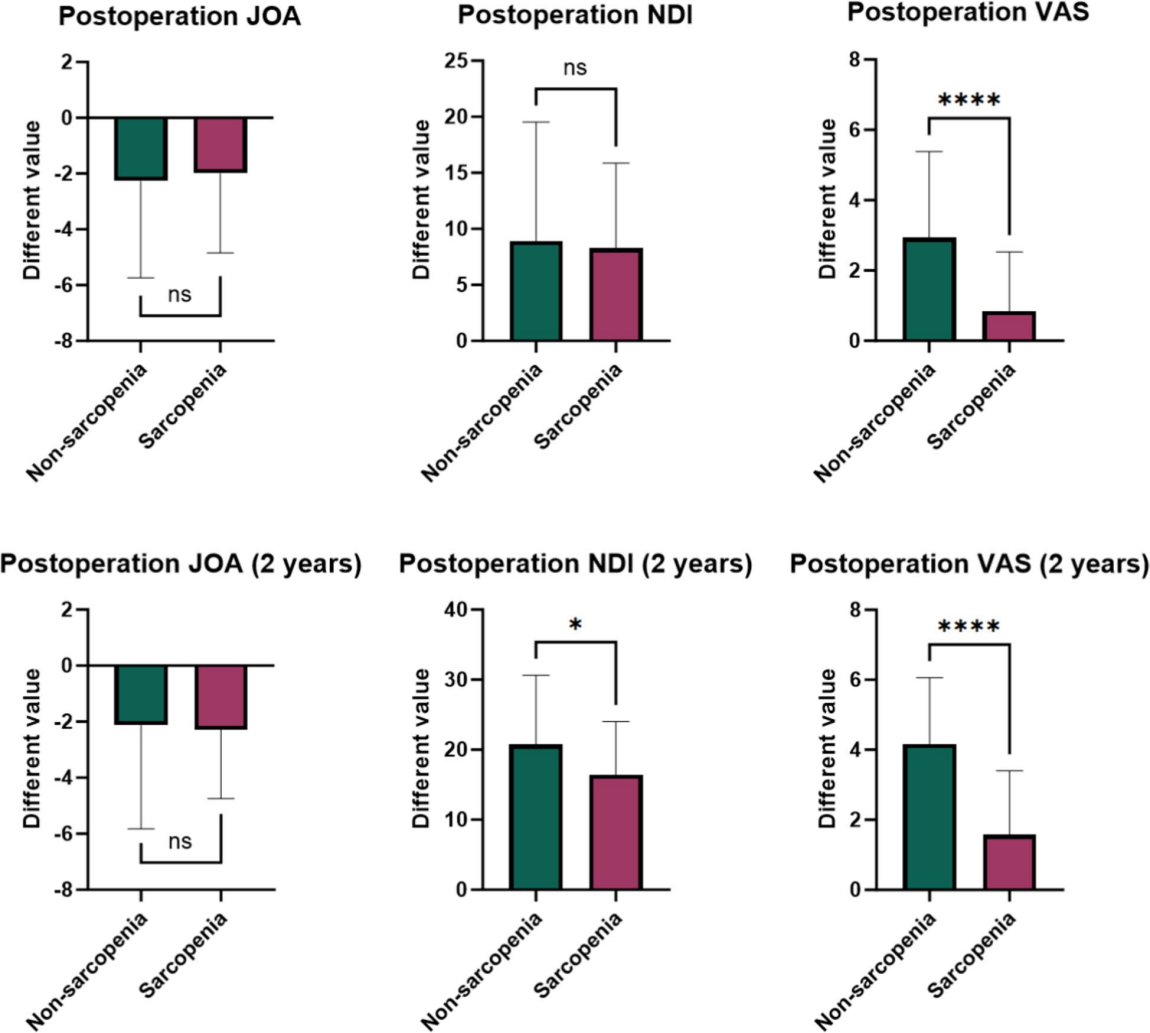
Comparison of improvements in JOA, NDI, and VAS scores between the two groups of patients (immediately postoperative minus preoperative, 2-year post-operative follow-up minus preoperative)

**Fig. 6:**
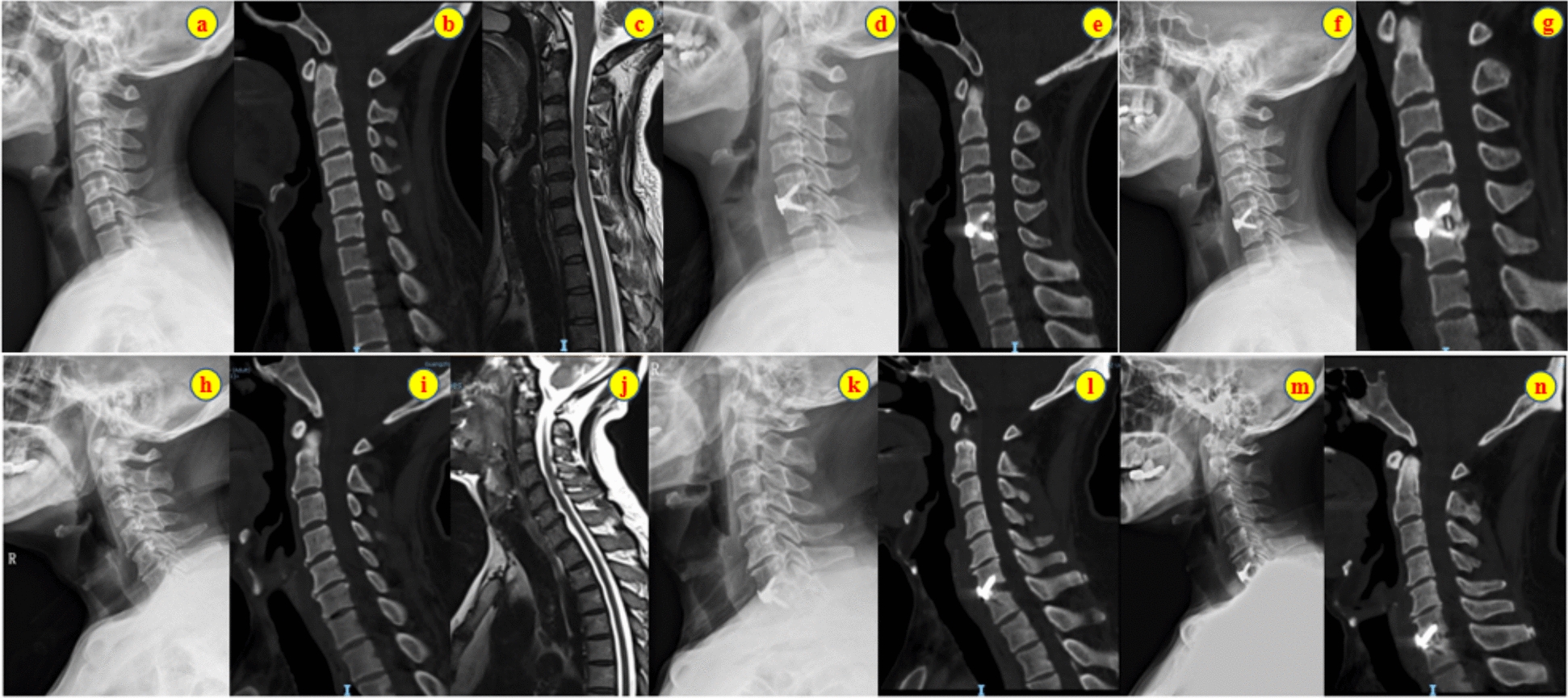
**a–g** Case of an elderly female patient without sarcopenia who underwent C5/6 ACDF surgery. **a–c** Preoperative *X*-ray, CT, and MRI examinations revealed cervical kyphosis and left-sided intervertebral foramen-type herniation at the C5/6 level; **d, e** Immediate postoperative *X*-ray and CT scans indicated stable fixation of the C5/6 internal fixation; **f, g** Two-year postoperative *X*-ray and CT scans revealed robust fusion at the C5/6 level. At 2-year postoperative, her symptoms improved significantly, with minimal discomfort. **h–n** Case of an elderly male patient with sarcopenia who underwent C6/7 ACDF surgery. **h–j** Preoperative examination revealed C6/7 intervertebral disc herniation posteriorly and centrally. **k, l** Immediate postoperative *X*-ray and CT scans revealed stable fixation of C6/7 internal fixation. **m, n** 2-year postoperative *X*-ray and CT scans indicated strong fusion at C6/7. Follow-up revealed residual radicular symptoms 2 years postoperatively

There was no statistically significant difference in the preoperative or immediately postoperative improvement in the JOA score or NDI score between the two groups immediately after surgery and at the 2-year follow-up (*P* > 0.05, Table 8); however, in terms of the improvement in the VAS score, the nonsarcopenia group not only exhibited superior improvements immediately after surgery compared with the sarcopenia group, but this advantage persisted at the 2-year follow-up (*P* < 0.05, Fig. 5). In addition, two typical cases further corroborate this finding: both the sarcopenia group and the nonsarcopenia group achieved stable fusion 2-year after ACDF surgery, but patients in the sarcopenia group still had residual radicular symptoms, whereas those in the nonsarcopenia group experienced almost no significant discomfort (Fig. 6).

**Table 8:** Intergroup comparison of improvements in JOA, NDI, and VAS scores (preoperative, immediate postoperative, and 2-year postoperative

	∆Score	Sarcopenia (*n* = 38)	Nonsarcopenia (*n* = 55)	*P* value
Post (Preop minus Postop)	∆JOA	− 2.00 ± 2.85	− 2.27 ± 3.47	0.690
	∆NDI	8.26 ± 7.61	8.93 ± 10.59	0.741
	∆VAS	0.84 ± 1.69	2.93 ± 2.46	< 0.001
Post 2 years (Preop minus Post 2 years)	∆JOA	− 2.29 ± 2.46	− 2.13 ± 3.70	0.814
	∆NDI	16.42 ± 7.59	20.75 ± 9.87	0.025
	∆VAS	1.58 ± 1.83	4.16 ± 1.90	< 0.001

## Discussion

ACDF has become the standard surgical procedure for treating CSM with surgical indications because of its advantages of direct decompression, restoration of the cervical intervertebral space height, and sagittal line of force [16]. However, clinical observations have shown that even when surgical decompression is adequate and internal fixation is optimally positioned, some patients experience incomplete pain relief postoperatively and suboptimal long-term recovery of cervical function. This suggests that, in addition to the surgical technique, the patient’s baseline condition may be a key factor influencing postoperative outcomes; sarcopenia is a potential influencing factor that has garnered significant attention in recent years [17].

Sarcopenia, a progressive, systemic skeletal muscle disease, has traditionally been defined in terms of a reduction in total muscle mass and muscle function. However, in the field of spinal disorders, changes in the quality of local muscles (such as those surrounding the cervical spine) are more clinically relevant: the cervical muscle group not only serves as a “biomechanical scaffold” for maintaining cervical spine stability but also directly influences the development and progression of spinal disorders and postoperative recovery by regulating cervical dynamic balance and participating in pain signal transmission [12]. Previous studies on degenerative diseases of the lumbar spine have confirmed that sarcopenia is associated with an increased incidence of postoperative complications and a decline in quality of life in patients [18]; however, for CSM patients, the impact of sarcopenia diagnosed on the basis of neck muscle mass on the postoperative outcomes of ACDF remains unclear, which is the core focus of this study.

This study included 93 patients with CSM (38 patients in the sarcopenia group and 55 patients in the nonsarcopenia group). There were no statistically significant differences between the two groups in terms of demographic characteristics, such as age, sex, BMI, surgical-related factors, such as surgical duration and intraoperative blood loss, or cervical spine anatomical and functional parameters, such as the preoperative OI and cervical ROM. This baseline balance has important clinical implications: on the one hand, it eliminates confounding factors such as age-related differences in surgical tolerance and preoperative cervical spine structural and functional abnormalities that could interfere with postoperative outcomes; on the other hand, it provides a prerequisite for attributing postoperative outcome differences between the two groups to sarcopenia, avoiding result bias caused by baseline imbalance and ensuring the reliability of the study conclusions.

Cervical sagittal plane parameters (e.g., T1s, C2‒C7 Cobb angle, ASDH) are core indicators for assessing structural recovery after ACDF surgery, directly reflecting the effectiveness and stability of cervical realignment [19]. This study found that all sagittal parameters in both groups improved significantly immediately postoperatively compared with preoperative values, and the degree of improvement did not differ statistically between the two groups.This finding eases the concern that sarcopenia may weaken spinal surgery’s structural corrective effects. The mechanism is closely linked to ACDF's surgical biomechanics: ACDF restores intervertebral disc height with a fusion device and maintains cervical sagittal alignment via internal fixation. This mechanical correction plays a dominant role, effectively offsetting the potential negative impact of reduced cervical muscle mass on cervical structural reconstruction [13, 20].

Notably, a previous meta-analysis of lumbar degenerative diseases indicated that sarcopenia has no significant impact on the structural outcomes of lumbar surgery [4]. This study extends this conclusion to the cervical spine domain—for the first time, it confirms that sarcopenia diagnosed on the basis of cervical muscle mass does not impair the corrective efficacy of ACDF on sagittal plane parameters in CSM patients. This finding provides important clinical guidance: for CSM patients with concomitant cervical sarcopenia, there is no need to be overly concerned with the structural reconstruction outcomes of ACDF, as surgery can still achieve optimal restoration of cervical spine alignment.

Compared with the consistency of structural indicators, the clinical function and pain outcomes of the two groups of patients exhibited the characteristic of “overall improvement with local differences,” which is one of the most clinically valuable findings of this study. Overall, both groups of patients showed significant improvements in JOA scores (neurological function), NDI scores (cervical function), and VAS scores (pain) immediately postoperatively and at 2 years post-operatively compared with preoperative levels, further validating the classic efficacy of ACDF in treating CSM—regardless of whether muscle atrophy is present, surgery can effectively alleviate spinal cord compression and improve overall patient function, which is consistent with the consensus in the field of spinal surgery regarding ACDF [21]. However, the key differences lie in pain relief and long-term functional recovery: the nonsarcopenia group had significantly better VAS scores (pain relief) immediately postoperatively (2.16 vs. 4.03) and at 2 years post-operatively (0.93 vs. 3.29) than did the sarcopenia group, with a mean between-group difference of 2.36 points in VAS at the 2-year follow-up. The nonsarcopenia group also demonstrated a clear advantage in NDI scores (long-term cervical function) at 2 years post-operatively (2.55 vs. 6.13), resulting in a mean between-group difference of 3.58 points; however, there were no differences in JOA scores (neurological function) or early postoperative NDI scores between the two groups. This “selective difference” can be explained by the physiological functional mechanisms of the cervical muscles: the muscles surrounding the cervical spine (such as the multifidus and longus colli muscles) not only serve as “mechanical stabilizers” for the cervical spine but also play a crucial role in maintaining the long-term stability of the corrected alignment after ACDF surgery [14]. Patients with sarcopenia experience a reduction in neck muscle mass, leading to insufficient muscle strength. Even if surgery achieves structural correction, postoperative residual pain or long-term functional compensation may still occur due to a decrease in the dynamic stability of the cervical spine [17, 22]. Moreover, neck muscles are involved in the peripheral regulation of pain signals. A reduction in muscle mass may lead to a decrease in the ability to clear local inflammatory factors and a decrease in the pain perception threshold, further exacerbating the postoperative pain experience [23, 24]. The results of the two typical cases presented in this paper provide a more intuitive confirmation of this mechanism: both groups of patients achieved stable fusion 2 years post-operatively, but the sarcopenia group still had residual radicular symptoms, whereas the nonsarcopenia group had no obvious discomfort. This suggests that the impact of sarcopenia on the postoperative outcomes of CSM patients following ACDF surgery is not reflected in whether “surgical structural fusion can be achieved” but rather focuses on whether “patients can achieve optimal symptom relief and long-term functional recovery”—this conclusion provides a clear direction for the development of postoperative rehabilitation protocols.

The innovation of this study lies in overcoming the limitations of traditional “whole-body assessment” of sarcopenia and, for the first time, the use of “neck muscle mass” as the core diagnostic basis to explore its impact on the postoperative outcomes of CSM patients undergoing ACDF surgery. Previous studies have mostly defined sarcopenia on the basis of limb muscle mass or whole-body muscle mass, which can reflect overall skeletal muscle status but are unable to precisely match the pathophysiological requirements of cervical spine-specific diseases [25, 26]. This study focuses on the anatomical characteristics of CSM and concentrates on the neck muscles as a “local functional unit.” The conclusions drawn are more closely aligned with those of clinical practice and fill the research gap regarding the association between local muscle atrophy and the outcomes of cervical spine surgery.

From a clinical perspective, the findings of this study offer two important insights. First, preoperative assessment of neck muscle quality in CSM patients (e.g., through CT/MRI quantification of neck muscle volume and fat infiltration) should be prioritized, as it serves as one of the predictive indicators for postoperative outcomes. For patients with concomitant neck sarcopenia, targeted intervention plans can be developed in advance (e.g., preoperative cervical muscle strength training and nutritional support to improve muscle quality). Second, postoperative rehabilitation should prioritize strengthening neck muscle function rather than solely focusing on restoring cervical spine mobility. By enhancing neck muscle strength, this approach can help patients with muscle atrophy alleviate postoperative pain and improve long-term neck functional stability.

This study has several limitations: first, sarcopenia diagnosis relied solely on imaging-derived neck muscle mass, lacking concurrent assessments of muscle strength (e.g., grip strength, neck extensor strength), quality (e.g., intramuscular fat infiltration rate), or function. This deviation from EWGSOP2 guidelines may compromise diagnostic accuracy and introduce misclassification bias. Second, the retrospective design—while balancing baseline characteristics (age, sex, BMI, surgical parameters) to reduce partial confounding—lacked systematic collection/control of confounders (smoking history, diabetes, nutritional status, postoperative rehabilitation compliance). This precludes precise isolation of sarcopenia’s independent effect via multivariate regression, potentially interfering with interpretations of VAS/NDI intergroup differences, and selection bias cannot be fully ruled out. Third, the small sample size may hinder detection of subtle outcome differences. Future prospective studies with larger samples should integrate imaging/functional assessments, include these confounders, and use multidimensional sarcopenia metrics (mass + strength + function) to establish a spine-specific definition, validating and quantifying its independent role in CSM postoperative recovery.

## Conclusion

In summary, sarcopenia, which is diagnosed on the basis of neck muscle mass, does not affect the structural correction of cervical sagittal plane parameters after ACDF surgery in patients with cervical spondylotic myelopathy, but it significantly weakens the degree of pain relief and the quality of long-term neck function recovery after surgery. These findings suggest that clinicians should focus on the functional foundation of the patient’s neck muscles while pursuing precision in ACDF surgical techniques. Through comprehensive management encompassing preoperative assessment, intraoperative protection, and postoperative rehabilitation, clinicians can strive to achieve optimal postoperative outcomes for CSM patients.

## Data Availability

The data used to support the findings of this study are included within the article.
